# Using the Multiple Analysis Approach to Reconstruct Phylogenetic Relationships among Planktonic Foraminifera from Highly Divergent and Length-polymorphic SSU rDNA Sequences

**DOI:** 10.4137/bbi.s3334

**Published:** 2009-11-11

**Authors:** Ralf Aurahs, Markus Göker, Guido W. Grimm, Vera Hemleben, Christoph Hemleben, Ralf Schiebel, Michal Kučera

**Affiliations:** 1 Department of Micropaleontology, Institute of Geosciences, Eberhard Karls University of Tübingen, Sigwartstraβe 10, 72076 Tübingen, Germany; 2 Organismic Botany, Eberhard Karls University of Tübingen, Auf der Morgenstelle 1, 72076 Tübingen, Germany; 3 DSMZ—German Collection of Microorganisms and Cell Cultures, Inhoffenstraβe 7 B, 38124 Braunschweig, Germany; 4 Department of Palaeobotany, Natural History Museum, Box 50007, 10405 Stockholm, Sweden; 5 Department of General Genetics, Centre of Plant Molecular Biology (ZMBP), Eberhard Karls University of Tübingen, Auf der Morgenstelle 28, 72076 Tübingen, Germany; 6 Laboratoire des Bio-Indicateurs Actuels et Fossiles, University of Angers, 2 bd Lavoisier, 49045 Angers, France. Email: ralf.aurahs@uni-tuebingen.de

**Keywords:** planktonic foraminifera, phylogeny, fossil record, automated alignment

## Abstract

The high sequence divergence within the small subunit ribosomal RNA gene (SSU rDNA) of foraminifera makes it difficult to establish the homology of individual nucleotides across taxa. Alignment-based approaches so far relied on time-consuming manual alignments and discarded up to 50% of the sequenced nucleotides prior to phylogenetic inference. Here, we investigate the potential of the multiple analysis approach to infer a molecular phylogeny of all modern planktonic foraminiferal taxa by using a matrix of 146 new and 153 previously published SSU rDNA sequences. Our multiple analysis approach is based on eleven different automated alignments, analysed separately under the maximum likelihood criterion. The high degree of congruence between the phylogenies derived from our novel approach, traditional manually homologized culled alignments and the fossil record indicates that poorly resolved nucleotide homology does not represent the most significant obstacle when exploring the phylogenetic structure of the SSU rDNA in planktonic foraminifera. We show that approaches designed to extract phylogenetically valuable signals from complete sequences show more promise to resolve the backbone of the planktonic foraminifer tree than attempts to establish strictly homologous base calls in a manual alignment.

## Introduction

DNA sequences coding for the 3’ segment of the small-subunit ribosomal RNA (SSU rDNA) have been broadly used to evaluate phylogenetic relationships among living planktonic Foraminifera.^1^–^13^ SSU rDNA data stored in international databases are in most cases sufficient to determine the systematic affinity of an unknown SSU rDNA fragment derived from a planktonic foraminifer using the blast algorithm.^14^ This is caused by two general characteristics of planktonic foraminiferal SSU rDNA sequences: (i) a higher intraspecific and interspecific variability in SSU rDNA regions which are generally conserved among most other foraminiferal lineages; and (ii) diagnostic sequences in SSU rDNA regions that are highly divergent between and among all major foraminiferal lineages.^8^,^15^,^16^ Those general characteristics nourished the hope that SSU rDNA data could be useful to address the evolutionary unfolding of all planktonic foraminifers.

**Figure 1.**
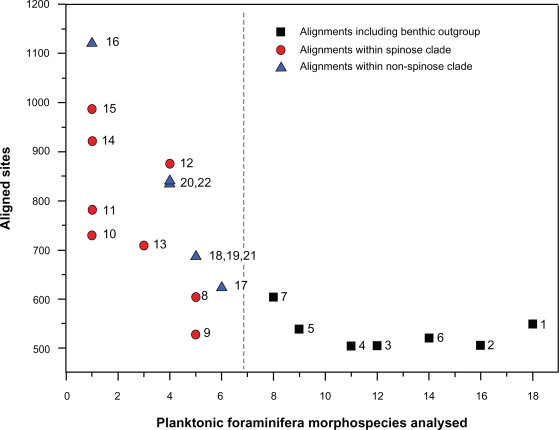
Lengths of manual alignments used to infer the phylogeny of planktonic foraminifera. Summary of planktonic foraminifera molecular phylogenies based on the 3’ fragment of the SSU rDNA gene. Almost one half of the ~1000 bp in the analysed fragment are lost when attempting to align “unambiguously” across the entire clade. The remaining variable regions clearly contain phylogenetically useful information, as can be seen by the longer alignments produced for subclades including only selected species. This phylogenetic information is lost when aligning across the three major clades of planktonic foraminifera, or when the alignment includes benthic outgroups. Data sources (in chronological order): 1997, Darling et al^2^ [**7**], Huber et al^4^ [**8**], de Vargas et al^3^ [**3**]; 1999, Darling et al^7^ [**5**]; 2000, Darling et al^9^ [**4**]; 2001, Stewart et al^11^ [**3**], de Vargas et al^10^ [**16,17**]; 2002, de Vargas et al^69^ [**9**]; 2003, Darling et al^70^ [**10,11,18**]; 2004, Darling et al^51^ [**19,20**]; 2006, Darling et al^54^ [**2,21**]; 2007, Darling et al^71^[**22**]; 2008, Kuroyanagi et al^72^ [**12**], Ujiié et al^73^ [**1**]; 2009, Aurahs et al^74^ [**13,14,15**].

However, phylogenetic inference has been hindered by the fact that the highly divergent SSU rDNA regions, which are of high taxonomic and phylogenetic value (Fig. 1), cannot be unambiguously aligned for all planktonic foraminifera. As a consequence, only up to 600 of the approximately 1,000 to 1,200 nucleotides of the more informative and thus commonly sequenced 3’ segment of the SSU rDNA have been used for phylogenetic studies of higher taxa in planktonic foraminifera (Fig. 1). In general, aligning noncoding sequences such as rDNA is more difficult than using protein-coding DNA fragments which are structured by reading frames and have most variability concentrated at third base positions within codons.^17^ Among foraminifera, this situation is aggravated by the fact that their SSU rDNA includes sequence strands (“expansion segments”) not found in the SSU of any other eukaryote up to now.^18^,^19^ These expansion segments are of unknown transcriptional fate, as the mature SSU rRNA of foraminifera has not been sequenced to date. Accordingly, any conclusions drawn from the reconstruction of the secondary structure have so far been of limited merits for inferring high-quality sequence alignments in this group of organisms. A further intricacy is that not all planktonic foraminiferal lineages exhibit the same level of sequence divergence from the common foraminiferal SSU rDNA motive. Nonspinose macroperforate and microperforate taxa in general yield SSU rDNA sequences which appear more similar to their benthic relatives than spinose taxa, as illustrated by short branches in phylogenetic trees and a generally low support for all backbone nodes. 5,7,9,11,20

In this situation, methods are urgently needed that avoid discarding phylogenetically valuable alignment positions but can also cope with the challenge of properly aligning those regions. In fact, the culling of alignment-ambiguous regions does not take into account that different possible alignment solutions do not necessarily imply different topologies or support values.^21^ Furthermore, exclusion of characters is often done on subjective grounds and further reduces the reproducibility of the results,^22^ which is frequently already low when an alignment is constructed manually, even if the secondary structure is considered. Consequently, Lee^21^ advocated an approach based on the generation of several alignments by the same algorithm but under different parameter combinations, which he called “multiple analysis method”. In multiple analysis, trees are inferred separately from the respective alignments and only relationships that are well supported in all (or most) of the trees are accepted.^23^Another possibility is to use different alignment algorithms under default values, as did Morrison and Ellis^24^ and Kemler et al.^25^ The latter approach appears to have several advantages; for instance, one would expect the differences between distinct alignment programs to be higher than those between different parameterisations of the same algorithm. That is, a larger proportion of the alignment space could be explored by running distinct programs. In addition, some parameterisations are simply not biologically reasonable, as, e.g. a scoring matrix that gives higher implicit weight to transversions than to transitions. Furthermore, current alignment algorithms and their default settings are constantly improved using benchmark tests (references for the individual programs are provided in Material and Methods below). Using the most recent version of the software out of the box, i.e. with default settings, is a straightforward approach to the sequence homology problem. In theory, sequence alignment cannot be considered separately from phylogenetic inference (e.g. many alignments programs use a guide tree), but both problems are NP-hard^26^,^27^ and in practice most researchers have regarded tree building as a distinct step (but see 28–30).

Despite the number of SSU rDNA sequences available, our knowledge of the actual diversity of planktonic foraminiferal SSU rDNA is still very limited (Table 1). Important taxa such as *Globorotalia*, including deep-dwelling species with relatively long reproductive cycles,^32^ *Globigerinita*, the to date only sequenced representative of the extant microperforate group, *Hastigerina pelagica*, the largest and morphologically most aberrant modern planktonic foraminifer, and most other spinose taxa save *Globigerinella siphonifera* and *Orbulina universa* are represented by single to few sequences in public databases.^4^,^6^ As a consequence, their genetic variability is not yet known to a sufficient degree. For about 20 planktonic foraminiferal species, i.e. half of the extant diversity in this group, no (reliable) sequence data are available yet (Table 1).

The collection of these species for DNA analyses from plankton samples has been hampered by their small size and relatively low abundance. The taxonomy (and classification; Table 1) of planktonic foraminifera is (still) based on the morphological characters of their calcite shells. Planktonic foraminiferal shells grow by sequential addition of proportionately larger chambers, typically along a trochospiral coil. The shape of individual chambers and the pattern of their addition can change considerably through ontogeny.^33^ Current taxonomic concepts are based on shells recovered from surface sediments. Such shells represent mature adult individuals that exhibit specific morphological characters. Living specimens afloat in the plankton, however, represent a range of mostly pre-adult ontogenetic stages that are lacking important taxonomic characters. Thus, it is possible that new, potentially extremely divergent SSU rDNA types will be found among not yet or not sufficiently sampled species, underscoring the need for phylogenetic approaches capable of objective and robust phylogenetic inference from divergent sequences.

In this study, we report new SSU rDNA data of planktonic foraminifera from the Azores Current System and the Mediterranean, including several new sequence types (Table 1). Our data is combined with the SSU rDNA stored in public databases (available until October 2008) and investigated using the multiple analysis approach as described above. This enables us (i) to combine the new and known planktonic foraminiferal SSU rDNA sequence types in reproducible approaches to phylogenetic analysis using all available sequence information in a time-efficient way, and (ii) to re-assess the phylogenetic relationships among planktonic foraminiferal lineages in comparison with earlier manual-alignment based work and evidence from the uniquely complete fossil record of these organisms.

## Material and Methods

### Sampling and DNA extraction

Live foraminifera in the Northwest Atlantic and the Mediterranean were sampled on RV Poseidon (P283/2, P308) and Meteor (M69/1) cruises using a multiclosing net (100 μm mesh size, sampling down to 700 m) and by filtering surface water from the ship’s uncontaminated seawater supply (65 μm mesh size). Specimens were isolated under an incident stereomicroscope (50-fold magnification), and taxonomically identified on board. After mechanical cleaning, single specimens were transferred to Eppendorff cups where the DNA was extracted following the DOC method from Holzmann and Pawlowski.^34^ Specimens were crushed in 50 μl of the DOC lysis buffer and incubated on a shaker table at 60 °C for one hour. Samples were than kept at −20 °C until PCR at the home based laboratory. Voucher information including the originally assigned morphotype and collection locality is provided in the Additional file 1.

### Data sources

#### GenBank data

SSU rDNA data of planktonic foraminifers were downloaded from the GenBank/NCBI taxonomy query portal (http://www.ncbi.nlm.nih.gov/; GWG, 28/10/2008).

#### Newly assembled data

Fragments of the 3’ SSU rDNA were amplified by PCR with Vent^®^ (New England Biolabs) polymerase using the primers S14f1,^8^ U/T20r1, U/A14f1,^35^ for later cloning and the new pelvF (5’TGACTCAACGCGG GAAATCT3’) and pelvR (5’CCGGGACATCTAAG GGCATCAC3’) primer pair for direct sequencing of few specimens of *Hastigerina pelagica*. PCR products were purified using the QIAquick gel extraction kits (Qiagen). Ligation and transformation relied on a pUC18/*E. coli* DH5α vector system. Genetic variability within single individuals was determined by sequencing up to five clones per individual and analysing PCR products obtained from several individuals per morphospecies where possible. Nucleotide sequencing was carried out in both directions with ABI 377 automatic sequencer (Perkin Elmer) using the standard vector primers M13uni and M13rev, or by a professional lab (Agowa, Berlin). The newly assembled SSU rDNA sequences have been uploaded to GenBank (accession numbers are provided in the Additional file 1).

### Alignments and phylogenetic inference

Multiple sequence alignments were inferred using six different software packages, clustalw version 2.0,^36^,^37^ kalign version 2.03,^38^ mafft version 6.24,^39^ muscle,^40^ the nralign derivative of muscle which uses an improved scoring function that considers neighbouring residues,^41^ and poa.^42^ clustalw was run either in default mode or with the gap opening and extension parameters optimized for RNA alignments (using the command-line switches-pwgapopen = 22.5 -gapopen = 22.5 -gapext = 0.83 -pwgapext = 0.83; henceforth referred to as clwopt).^43^ mafft was applied with the command-line switch-maxiterate 1000 and either default settings otherwise (henceforth called mafft), -localpair (linsi), -genafpair (einsi) or -globalpair (ginsi). poa was run in both default and global scoring mode (applying the command-line switch -do_global; henceforth referred to as poaglo) using the blosum80_trunc. mat substitution matrix delivered with the software and extended to include the complete nucleotide ambiguity code (the matrix is contained in Additional file 2). Accordingly, a total of eleven alignments were examined (included in Additional file 2).

Phylogenetic trees were inferred from the eleven alignments (without further processing such as a manual re-alignment or manual exclusion of sites) under the maximum likelihood (ML) criterion with RAxML version 7.04.^31^,^44^ RAxML has been specifically designed to efficiently handle large to extremely large datasets and infers phylogenetic trees with ML values at least as large as comparable contemporary programs. To establish node support, we used RAxML’s novel fast bootstrap option and 100 replicates in conjunction with the GTRMIX option (command-line switches -m GTRMIX -f a -# 100). GTRMIX applies the fast and memory-efficient GTRCAT model approximation during tree search but estimates the final log Likelihood and branch lengths under GTR + GAMMA.^31^,^45^ The fast bootstrapping has been shown to result in values close to standard bootstrapping, but also in an approximately ten-fold increase in performance.^44^ RAxML automatically infers a globally best (best-known) ML tree from the individual bootstrap trees in this running mode.

In the case of alignment-ambiguous data, the effects of different underlying alignment algorithms on phylogenetic reconstruction are usually greater than the effect of the different inference methods.^24^ Therefore, one might argue that it is sufficient to apply only the consistent and robust maximum likelihood (ML) criterion to infer phylogenetic trees. Nevertheless, to assess the effect of applying another phylogenetic optimality criterion, we calculated bootstrap support under maximum parsimony (MP) with PAUP* version 4b10.^46^ For each of the 100 bootstrap replicates, 10 random sequence addition replicates were conducted, saving only one tree per run. To compare the methods, MP support values were mapped on the corresponding ML trees for each alignment (Additional file 2).

For displaying bootstrap support values, we identified the most representative of the eleven best ML trees inferred from the distinct alignments. This was done by calculating all-against-all Robinson-Foulds distances between the best trees using PAUP* version 4b10 and determining the tree with, on average, the smallest distances to each of the other trees.^46^,^47^ The Robinson-Foulds distance between two trees is defined as the sum of the number of splits (bipartitions) present in one tree but not in the other. Support values from all bootstrap runs were mapped on the most representative tree using RAxML’s -f b command-line switch and integrated in one tree file using a UNIX shell script written by MG. For the trees, we also reported the final estimate for the alpha value of the gamma distribution and the log likelihood values of the best trees inferred with RAxML.

In order to quantitatively compare the alignments, we determined their total length. We additionally classified them using the alignment comparison metric (overlap score) as implemented in mumsa version 1.0,^48^ which also infers UPGMA dendrograms from these similarity values. A corresponding UPGMA classification of the RAxML trees was inferred from their Robinson-Foulds distances with PAUP*.^46^,^47^ To quantify the agreement of the phylogenetic trees with the current taxonomy of planktonic foraminifers, the affiliations of sequences to species were coded as a multi-state pseudocharacter (with one character state per species) for use under the maximum parsimony criterion.^49^,^50^ Newly obtained sequences from undetermined specimens and GenBank accession lacking a valid species name in their organism entry (e.g. “Orbulina sp. ‘isolate A102’ ”) were coded as missing data. The parsimony score of each of the best ML trees under this matrix (which we call „T-score“) was determined with PAUP*, higher scores indicating lower agreement. The pseudocharacter matrix is contained in Additional File 2.

## Results and Discussion

### Comparison of multiple sequence alignments

**Figure 2.**
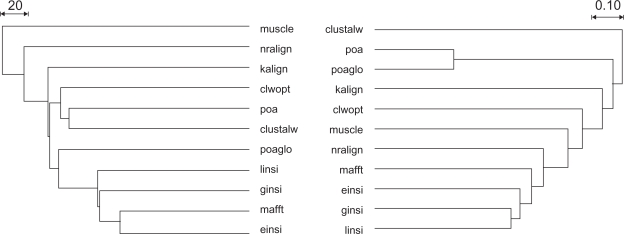
Comparison of alignments and trees. UPGMA dendrograms inferred from overlap scores between sequence alignments (right) and from Robinson-Foulds distances between the corresponding trees (left) are shown. Based on this comparison, einsi, ginsi and linsi were not considered further because they are too close to the mafft approach. muscle and clwopt were omitted because they resulted in some sequences being severely misplaced (see text). Apparently, tree topology can partially (mainly the close relationship of einsi, ginsi, linsi and mafft) be predicted by the comparison of the underlying sequence alignments.

The features of the inferred alignments and ML trees are shown in Table 2. Considerable differences regarding alignment length, estimated alpha values of the gamma distribution and highest obtained likelihood values were observed. This is in accordance with the prediction that the use of different alignment programs, instead of using a single software under a range of parameters, is sufficient to cover a large proportion of the alignment space. Here, clustalw results in the shortest SSU rDNA alignment and muscle in the longest. Classifications of the eleven approaches based on the alignments as well as the inferred trees are shown in Figure 2. The relationships indicated by the Robinson-Foulds distances between the best ML trees do not exactly mirror the relationships between the alignments as measured using the overlap score. For instance, the poa and poaglo alignments are similar to each other (Fig. 2, right), but the poa-based ML tree is more similar to the clustalw-based trees than to the poa-glo-based tree with respect to Robinson-Foulds distances (Fig. 2, left). On the other hand, the mafft-, einsi-, ginsi- and linsi-based trees are clustering together, as do their underlying alignments. Our observations on alignment and topological comparison measures are important for future multiple analysis studies as far as they indicate that the shape of the tree cannot always be predicted from the descriptive characteristics of the alignment, at least in the case where highly divergent sequences are considered.

**Figure 3.**
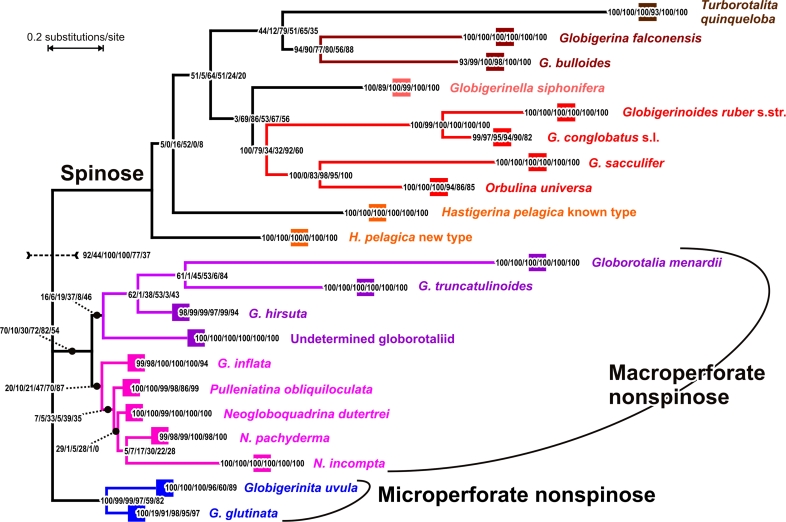
Partly collapsed ML tree inferred from the MAFFT alignment. The best ML tree inferred from the mafft alignment is shown. Branches are scaled in terms of the expected numbers of substitutions per site. Subtrees that include only sequences from the same morphospecies are collapsed at their root node and represented by black rectangles. Support, i.e. bootstrap percentages from the clustalw/kalign/mafft/nralign/poa/poaglo-based analyses, of the collapsed subtrees and their relationships is indicated on the terminal nodes and on the branches. Not collapsed and accordingly annotated versions of all best known trees are found in the Additional file 2.

Regarding the agreement with morphotaxonomy, the best (minimal) T-score observed is 23, obtained by nine of the eleven alignments (Table 2). This again is in agreement with the prediction that the use of alignment programs under default values, instead of using a single software under a range of parameters, results in biologically reasonable alignments that do not contradict previous taxonomic knowledge. The fact that even the best obtained T-scores are three steps larger than the minimum possible score of 20 (corresponding to 21 pseudocharacter states) is caused by three mislabelled sequences, whereas scores higher than 23 are due to misaligned sequences (shown below). Thus, trees inferred from muscle and clwopt achieving T-scores of 25 were not further considered for displaying trees and drawing conclusions on foraminifer evolution. The particularly low likelihood observed for the muscle tree could also be caused by one to several sequences being severely misaligned. However, the likelihood of the best tree cannot directly be used to select the best alignment, because common ML functions, as those implemented in RAxML, do not consider gaps. Also, einsi, ginsi, and linsi were not considered further because they were too close to mafft regarding both alignment and topological similarity (Fig. 2). ML bootstrap results from the six selected alignments were mapped on the mafft tree (Fig. 3), which was the most central one (the least distant from all other trees), irrespective of whether einsi, ginsi, and linsi were considered or not.

A comprehensive table of well-supported (ML/MP) and/or systematically relevant phylogenetic splits is provided as supplement (Additional file 3); all alignments and trees are included in Additional file 2. In general, ML and MP support the same phylogenetic splits (bipartitions), although the support under MP is often lower than under ML using the same alignment. At the species level or higher, ML supports 23 bipartitions with high support based on all six alignments (BS_ML_ ≥ 80), and four more if only five out of the six alignments are considered. Using MP as optimality criterion 22 bipartitions are highly supported based on all six alignments, and an additional one based on five out of six alignments. In all remaining bipartitions, high ML bootstrap support correlates to moderate MP bootstrap support. Only two exceptions were observed: In one case, kalign-based ML bootstrap support is low (BS_ML_ = 12), and MP high (BS_MP_ = 100). In the other, the situation is vice versa (BS_ML_ = 89; BS_MP_ = 12). In both cases, short sequences are involved. It appears that the portion of missing data, in combination with the kalign-generated alignment, can negatively affect ML and MP inferences, but has little effect elsewhere.

### SSU rDNA sequence diversity in planktonic foraminifera, and misidentified or unidentified specimens and sequences

As stated in the introduction the identification of plankton material is challenging and often leads to ambiguities in species determination. This is reflected in several mislabelled sequences found in online databases but also in our collections. The comprehensive evaluation of all database sequences in the course of our study reveals that one Gen-Bank sequence has been mislabelled (Z69600; in GenBank stored as *Globigerinoides sacculifer*, but obtained from a *G. conglobatus* individual^6^) and that the single sequence of *Globorotalia crassaformis* stored in GenBank (AY453134) is 100% identical to sequences of *G. inflata* considering the amplified fragment (newly assembled and public database data). The single *Globigerinella calida* accession (Z83960) is identical to one SSU rDNA type of *G. siphonifera* (Additional files 2, 3). Considering the general level of SSU rDNA divergence within and among morphospecies detected elsewhere (this study,^6^,^7^,^9^,^51^) it is likely that these database sequences have been misidentified on collection, although currently no comparative data exist for *Globorotalia crassaformis* and *Globigerinella calida.*

In our new dataset, two clones of a newly sampled *Globigerina bulloides* specimen (R043) are showing sequence types characteristic for, and well documented in, *Globigerinella siphonifera.* These sequences were placed in all ML trees within the *G. siphonifera* clade. Together with Z69600, the R043 clones were responsible for the best T-scores being three steps larger than the minimum possible score (23 vs. 20). Accordingly, all alignments which resulted in a best ML tree achieving a T-score of 23 were regarded as in agreement with morphotaxonomy (the singletons AY453134 and Z83960 do not have an effect on the T-score of distinct topologies); the two exceptions were clwopt and muscle. In trees inferred from the muscle alignment, one (incompletely sequenced: 436 bp) *Globigerinita glutinata* clone (R04903) was placed within *Neogloboquadrina dutertrei*. Trees inferred with clwopt even misplaced four *Globigerinita glutinata* sequences (R04903, R04906, R049a1, and AF250105) within *Neogloboquadrina pachyderma*, apparently also an artefact caused by short sequences.

In addition to the identification of mislabelled sequences, ca. 20 sequences in our new dataset obtained from small specimens that could not be properly determined (R021, R034, P155, P125), and gene bank accessions labelled “*Globigerina* sp.”, were unambiguously placed in all trees; they nested within existing clades that received high support (Additional file 2). These sequences thus could be identified by their position in the phylogenetic reconstructions and have been treated accordingly for the following discussion.

### Monophyly of morphospecies

Figure 3 depicts a reduced ML tree inferred from the mafft-generated alignment, together with boot-strap support (BS_ML_; bootstrap percentages based on 100 replicates) for individual nodes inferred from six selected alignments. For the sake of simplicity, subclades referring to distinct morphotaxa have been collapsed; full, annotated trees can be found in the Additional file 2. Tables 3 and 4 list in addition the bootstrap support of respective bipartitions under MP (BS_MP_); further details can be found in Additional file 3.

Most terminal nodes received high support from the bootstrap analyses (BS_ML/MP_ > 80) independent of the alignment and inference method used; these are the nodes that define molecular clades corresponding to morphologically defined species (Fig. 3; Table 3). Exceptions were *Globigerinita uvula* (BS_ML/MP_ = 60/29, poa; BS_MP_ = 59, poaglo; BS_ML/MP_ ≥ 89, others) and *Hastigerina pelagica*. The latter forms a low (under MP) to moderate or high (under ML) supported clade only in the poa-based and poaglo-based analyses (Table 3). In two cases ML and MP bootstrap support differs strongly as inferred from the kalign alignment (*Globigerinita uvula; Globigerinella siphonifera*). This is likely due to short sequences which are not optimally aligned by this software (see above).

The GenBank sequence of *Globigerinita uvula* (AF387173) is markedly different from other SSU rDNA sequences of planktonic foraminifers in the expansion segments (not shown, but see Additional file 2). Before this study, three sequences have been documented from its nearest relative, *G. glutinata*. We could amplify SSU rDNA fragments from two small individuals, which were identified upon collection as juveniles of either *Turborotalita quinqueloba* or *Globigerinita uvula*. We obtained and sequenced five clones from these two individuals documenting a new genotype comprising two similar sequence variants (details not shown). This genotype is placed as sister clade to the single *G. uvula* sequence from GenBank (BS_ML/MP_ between 59 and 100; except based on the poa-alignment), and both are placed as a sister clade to *G. glutinat*a (Fig. 3; Table 4). We therefore assume that the collected specimens comprise a new sequence type of *G. uvula*. However, it is clear that this group requires much more attention and data (see Table 1).

The most unexpected result of our survey of sequence diversity among the Azores Front planktonic foraminifera was the discovery of a new and highly divergent sequence type isolated from specimens of *Hastigerina pelagica.* Until now, this morphospecies has been represented by a single sequence in the public databases (Z83958;^6^). For this study we had access to SSU rDNA data from eleven specimens of *H. pelagica*, and a total of 38 sequences, mostly clones but also directly sequenced PCR products. Two of these specimens yielded a sequence type consistent with the template Z83958; the remaining nine specimens yielded the new type. The two types differ markedly in their nucleotide sequences (cf. length of the root and placement of both types in Fig. 3). In the ML trees inferred from four of the six alignments, the two sequence types of *H. pelagica* were placed in a grade-like fashion at the root of the spinose group with diminishing support (Fig. 3; refer to Additional file 3 for BS_MP_). In trees from the POA and poaglo alignments, *H. pelagica* formed a clade with high to moderate support under ML but not MP (see above; Table 3); and this relationship received little support otherwise (Table 3). None of the alternatives received a considerably higher support than any other based on all six alignments and both optimality criteria (Additional file 3). Thus, our analysis is inconclusive considering the position and relationships of both *H. pelagica* types.

The Hastigerinidae exhibit several morphologically unique features, including triradiate spines, mono-lamellar shell and a peculiar cytoplasmic “bubble capsule”.^32^ *Hastigerina pelagica* is one of the easiest identifiable extant species of planktonic foraminifera and a misidentification of the individuals yielding one of the two SSU rDNA genotypes can be largely ruled out. The only other two members of the family Hastigerinidae are *Hastigerinella digitata* and *Orcadia riedeli* (Table 1), which can be distinguished from the latter by chamber shape and spines distribution.^52^ With regard to the unique morphology of *H. pelagic*a and considering the morphological variability among other spinose taxa,^32^ it also appears unlikely that these characters have evolved in parallel and that they would be indicative of anything else than a common origin. On the other hand, the available SSU rDNA data do not support any scenario that would strongly contradict a common origin of *H. pelagica* (Additional file 3). One explanation why molecular data do not support a monophyly of *H. pelagica* (Table 3) might be a deep divergence followed by a rapid radiation.^53^ This situation is analogous to that of *Neogloboquadrina incompta*—*N. pachyderma.* Both species differ only in their preferred coiling direction and have been traditionally placed in one species, *N. pachyderma.*^54^ Like *H. pelagica* this pair is represented by divergent sequence types not supported as sister taxa in phylogenetic trees (Fig. 3; Table 4;^9^,^51^ using limited taxon samplings).

This analysis, like previous work, largely supports the monophyly of SSU rDNA sequences from currently accepted and analysed morphospecies of planktonic foraminifera.^13^,^55^ Save *H. pelagica* as outline above, there is one more exception to this rule, namely the biphyletic nature of sequences collected from specimens identified as *Globigerinoides ruber*. Two main SSU rDNA genotypes have been reported from the white variant of this species, one (“Type II”)^7^ being placed as a sister taxon to *G. conglobatus* (the clade here referred to as *G. conglobatus* s.l.);the other(“Type Ia”, “Ib”) forming a distinct clade with the pink-pigmented variant (here referred to as *G. ruber* s.str.; following the common notion that species should mirror monophyla).^7^ All analyses have recovered this relationship: Both the *G. conglobatus* s.l. and the *G. ruber* s.str. clades obtained comparably high to very high support (BS_ML/MP_ ≥ 82 and BS_ML/MP_ = 100, respectively; Fig. 3, Table 3). The sister group relationship of the two clades was highly supported (BS_ML/MP_ ≥ 99) in trees from all six selected alignments (Fig. 3; Table 4).

### Interclade relationships

Several relationships depicted in the mafft-inferred ML tree (Fig. 3) were consistently recovered by all methods. The mutual monophyly of each of the three major lineages of planktonic foraminifera recognized on the basis of their shell ultrastructure,^32^ i.e. the microperforate nonspinose, the macroperforate nonspinose, and the spinose groups, was moderately to well supported under ML as the optimality criterion (Fig. 3; Table 4). Support under MP of such ‘deep’ relationships is, however, markedly decreased (Table 4; see also Additional file 3 for other ‘deep’ relationships; Additional file 3). An explanation may be that MP becomes statistically problematic, if the rate of change is high.^56^

As noted in the introduction, this is the first comprehensive (full) analysis of SSU rDNA data of planktonic foraminifera since the work of de Vargas et al.^3^ That study used 521 “unambiguously aligned” sites among 15 morphospecies and the trees were rooted on several benthic foraminifera species (seven in total, including monothalamids and polythalamous taxa) as outgroups. The analyses identified the same three major planktonic groups, and as in our study, with varying support from nonparametric bootstrapping under different optimality criteria (low to high, a single sequence included representing the microperforate group; Table 4). There have been several later attempts that also included data from all three major lineages (Table 4). They partly found moderate to high support (Table 4) using only the conserved (“unambiguously alignable”) sites of the 3’ SSU rDNA, however, at the cost that not all SSU rDNA data-covered taxa were included. In the light of the arbitrarily restricted taxon sampling of these studies, they can neither be straightforwardly compared with the results of de Vargas et al^3^ nor with this study. From a qualitative point of view, our study agrees with all former analyses in their separation of the three major groups of planktonic foraminifera (but see^7^). Since our focus here was to evaluate the multiple analysis approach to infer a phylogenetic structure *within* planktonic foraminifera and not to place planktonic taxa in an all-foraminiferal phylogeny, we did not include any benthic group. Nevertheless, it could be interesting to see, where the planktonic lineages will be placed in analyses based on matrices, which include *all* available SSU rDNA data of foraminifera.

In addition to relationships recovered by de Vargas et al^3^ (morphotaxa generally forming clades, recognition of a macroperforate and spinose clade; microperforate representative distinct from other planktonic foraminifera; a *G. conglobatus—G. ruber* clade; Table 4), some more interspecific relationships can be found, which are addressed in more detail in the following.

#### The microperforate nonspinose clade

Our analyses include data from two (or possibly three) morphospecies of *Globigerinita*. Their monophyly (distinctiveness) is well supported (Fig. 3; Table 4; poa-based moderate support). Up to now there has been no comprehensive study using the SSU rDNA data of *Globigerinita* (but see^3^).In one earlier analysis, data from both species was included.^11^ The distance-based reconstruction used 505 sites from the generally conserved parts of the 3’ SSU rDNA. As a result the planktonic lineages were placed along an unresolved polytomy with various benthic taxa. It has to be noted that only two nonspinose taxa were included (*Neogloboquadrina dutertrei* and *N. incompta*) and most of the inferred nodes were unsupported (Table 4).

#### The macroperforate nonspinose clade

**Figure 4.**
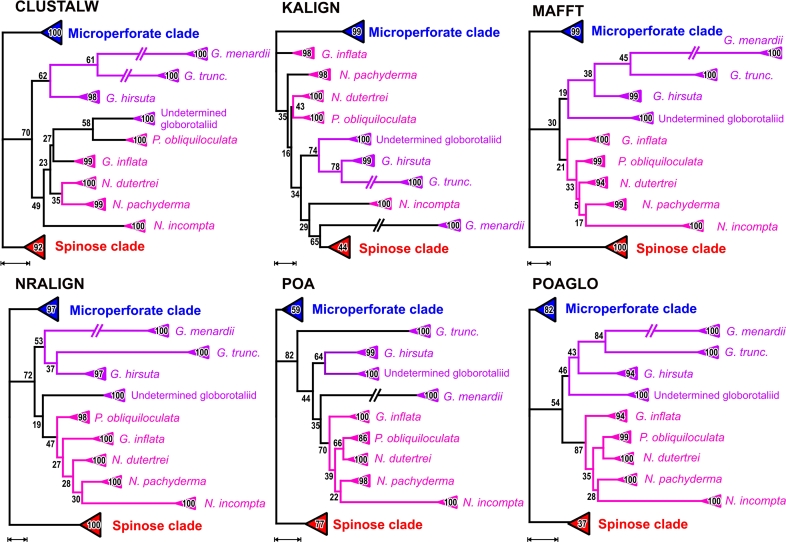
Alternative phylogenetic relationships within the nonspinose macroperforate clade as inferred from the six alignments. Shown are reduced ML phylograms based on the six selected alignments, with bootstrap support under ML annotated on the according branches (MP bootstrap support can be found in Additional files 2, 3). Scale bars are adjusted to 0.1 expected substitutions per site. Where indicated, branches have been broken down to one half of the original length. Subtrees comprising the same morphospecies were collapsed, as in Figure 3, as well as the microperforate (blue triangle) and spinose (red) clades. Not collapsed full ML trees can be found in the Additional file 2.

The multiple analysis approach reveals no consistent phylogenetic structure within the macroperforate group, with support for individual nodes being generally low (Fig. 4; see also Additional file 3). *Globorotalia inflata* tends to group with the Neogloboquadrinidae unlike the other *Globorotalia* species (Fig. 4). This result is comparable to culled-alignment analyses of SSU rDNA,^3^,^10^ the only two other studies that used data of all nonspinose taxa that were available at that time. Darling et al,^51^ reporting on evolutionary relationships within the Neogloboquadrinidae (*Neogloboquadrina* spp.*, Pulleniatina obliquiloculata*), used *Globorotalia inflata* as an outgroup, because it could be better “unambiguously aligned” with the former than the other globorotaliids (685 sites).^51^ This is, however, not quantifiable based on the multiple analysis results. Any alternative of inter-specific phylogenetic relationships within the non-spinose clade received diminishing support, both under ML and MP (but see Additional file 3 considering the putative sister pair *N. dutertrei—P. obliquiloculata*; Table 4).

#### The spinose clade

**Figure 5.**
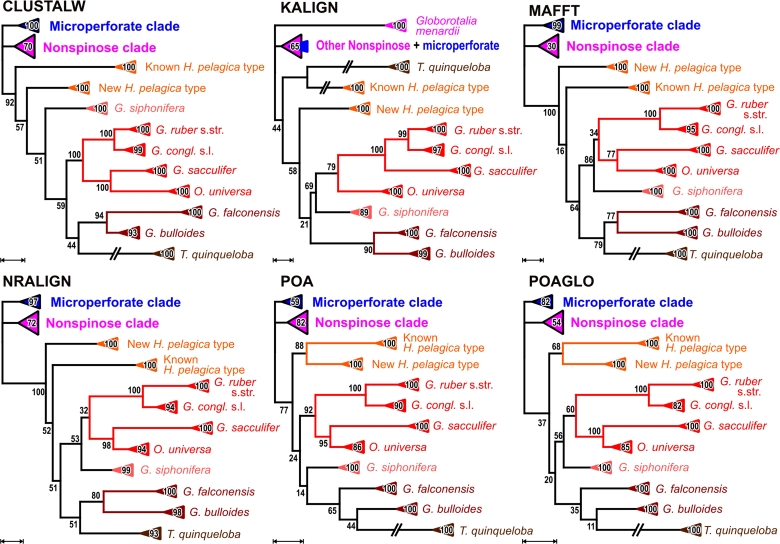
Alternative phylogenetic relationships within the spinose clade inferred from the six alignments. Shown are reduced ML phylograms based on the six selected alignments, with BS_ML_ annotated on the according branches. Scale bars are adjusted to 0.2 expected substitutions per site. Where indicated, branches have been broken down to one half of the original length. Subtrees comprising the same morphospecies as well as the microperforate and nonspinose macroperforate clades were collapsed, analogous to Figures 3 and 4. Not collapsed full ML trees can be found in the Additional file 2.

Despite the higher divergence among the spinose lineages, several relationships were consistently recovered by most or all of the analyses (Figs. 3 and 5). A *Globigerinoides conglobatus-G. ruber* clade received the highest support (BS_ML/MP_ ≥ 99; Fig. 5; Table 4), and has also been found in all former studies based on filtered SSU rDNA data.^3^,^7^,^9^,^11^ The sister clade of *G. conglobatus-ruber* comprised *Orbulina universa* and *G. sacculifer* implying a common origin of these four morphospecies; this clade was represented in all six ML trees with BS_ML_ between 32 and 100 (Fig. 5; Table 4). As for the major clades (microperforate, nonspinose macroperforate, and spinose clade; Fig. 3), bootstrap support of this relatively ‘deep’ relationship is markedly lower under MP than under ML (Table 4). In five of the six analyses *Orbulina universa* appeared as sister group of *G. sacculifer* (BS_ML/MP_ ≥ 82; Fig. 5; Table 4). Similar relationships have been reported although with low (<50) bootstrap support (Table 4) using filtered SSU rDNA data and distance-based reconstructions (neighbour-joining).^7^,^9^,^11^ In the more comprehensive study of de Vargas et al,^3^ *G. sacculifer* and *O. universa* formed a low to moderately supported clade with *Globigerina bulloides* under ML, distance and parsimony (Table 4).

*Globigerina bulloides* and *G. falconensis* were supported as sister taxa by bootstrap analysis (BS_ML/MP_ ≥ 53; Fig. 3; Table 4). They were, however, placed as grade in the poa- and poaglo-based ML trees (Fig. 5), with *G. bulloides* placed as sister taxon to *Turborotalita quinqueloba*. Such a topology received generally less support than the alternative of *Globigerina* clade (Fig. 3; poa-based ML tree provided in Additional file 2). This underscores the importance of establishing and investigating support (here: nonparametric bootstrapping) in course of multiple analysis (Figs. 3–5; Tables 3, 4), rather than to focus on clades found (or not) in the inferred phylogenetic trees (Figs. 4, 5). A one-alignment-one-tree approach may fail to recover an otherwise supported relationship unless the bipartition tables are investigated, because it is not represented in the inferred tree.

The placement of the extremely long-branched *T. quinqueloba* remains ambiguous. The support for a common origin of *Globigerina* and *Turborotalita* ranges from very low (kalign) to moderate (mafft, nralign, poa; Figs. 3, 5; refer to Additional file 3 for BS_MP_). A sister relationship between *T. quinqueloba* and *G. bulloides* has been found in distance-based analyses,^9^,^11^ which are prone to long-branch attraction more than ML.^56^,^57^ As one alternative, *T. quinqueloba* was placed as sister clade to the known *Hastigerina pelagica* type (kalign), which is the longer branching of both *H. pelagica* types. *Hastigerina pelagica* has not been included in most traditional reconstructions that relied on filtered data, except in de Vargas et al.^3^ At the time of de Vargas et al,^3^ no SSU rDNA data of *T. quinqueloba* was available.

The last spinose taxon to be grouped within the spinose subtree is *Globigerinella siphonifera.* This taxon is placed by four of six alignment methods as a sister to the *Globigerinoides-Orbulina* clade, the according bipartition is moderately supported under ML by five of six alignments (BS_ML_ between 53 and 86; Fig. 5; Table 4). As in the case of the mutual monophyly of the three major groups, a common origin of *Globigerinella* and *Globigerinoides* + *Orbulina* finds support under ML as optimality criterion, but not if MP is used (BS_MP_ ≤ 26). Alternatively, this clade is placed as sister to the *Globigerina*-*Turborotalita* clade (poa-based; very low BS under ML and MP); or sister of all spinose taxa except *Hastigerina* (clustalw-based; BS_ML/MP_ = 51/24; BS_ML/MP_ ≤ 5 other; Fig. 5). Based on filtered SSU rDNA data, the position of *G. siphonifera* within the spinose clade remained essentially unresolved (3,7,9,11 , but see^5^).

### Comparison with the fossil record

The calcite shells of planktonic foraminifera accumulate in huge quantities on the sea floor, and in deep-sea basins they are a significant constituent of the sediment. The fossil record of planktonic foraminifera is one of the most complete and continuous of all organisms. Most significantly, the palaeontological taxonomy of this group is consistent with that of the living species, as both are based exclusively on the characters of the mineral shell. Because of the rich and continuous fossil record, phylogenetic relationships among fossil lineages of planktonic foraminifera are typically resolved by the method of stratophenetic tracing (58, among others). Here, the morphology of individual species is traced back through time in short temporal steps until the time of its first appearance, and the ancestor is then determined by tracking of intermediate morphologies at higher temporal resolution. It is important to note that the reconstruction of the phylogeny of the modern species has rarely been the main aim of detailed palaeontological investigations and that many of the phylogenetic relationships remain obscure, but could potentially be linked to the fossil record when appropriate effort and methods were applied.

**Figure 6.**
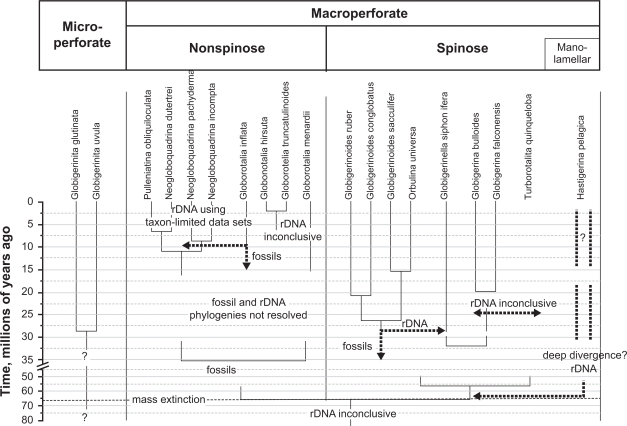
Comparison to the fossil record. A compilation of the fossil record of modern lineages.^59^,^60^,^64^ Solid lines represent known fossil ranges of species or lineages leading to these species. Incongruence between the molecular-based hypothesis and the fossil record is highlighted; fossil evidence that is contradictory to molecular phylogenies but poorly resolved is also indicated.

A synopsis of the multiple analysis results (superspecific clades) and our interpretation of the underlying data together with a schematic compilation of the fossil record of the analysed taxa are shown in Figure 6. Relationships of planktonic foraminifera, which appear well resolved in the fossil record, are included in Table 4, together with a summary of the support given by previous phylogenetic studies and multiple analysis under ML and MP. The characteristics of the wall structure of planktonic foraminiferal shells proved to be highly conserved through time (e.g. there have never been any microperforate foraminifera with spines and none of the spinose lineages is known to have lost spines) and support the existence of three main groups,^59^,^60^ which also find support in SSU rDNA sequence analyses (de Vargas et al^3^ and this study). The macroperforate spinose and nonspinose groups are considered to have shared a common ancestor in the Cretaceous—Paleocene genus *Hedbergella*.^7^,^59^,^61^ The earliest spinose species is considered to have evolved from *Hedbergella monmouthensis*, one of the few survivors of the Cretaceous-Tertiary extinction.^62^ However, the transition from the nonspinose to spinose state has never been observed, indicating that it must have been a rapid event associated with the filling of planktonic niches vacated after the mass extinction. Such an ancient and rapid divergence may not leave a conclusive signal in the genes of modern descendants,^53^ as mentioned in the case of the two divergent types of *Hastigerina pelagica*. The (common) ancestry of the macroperforate nonspinose group is less well constrained, but the hypothesis presented in Pearson et al^60^ links this group with another survivor species of the Cretaceous-Tertiary extinction, *Hedbergella holmdelensis*. The divergence between the two groups would thus be dated to the latest Cretaceous, 70–65 million years ago.

The most likely ancestor of the modern microperforate planktonic foraminifera is the genus *Guembelitria*, a survivor of the Cretaceous-Tertiary extinction which possessed a microperforate wall texture,^52^ although it must be noted that the link between the modern *Tenuitella* and *Globigerinita* forms and the Paleocene progeny of the *Guembelitria* lineage remains unresolved.^59^,^60^ This fossil-based phylogenetic hypothesis implies that the modern micro-perforate foraminifera represent a monophyletic clade, which is distinct from both the spinose and nonspinose macroperforate lineages. The origin of the Guemblitriidae in the late Cretaceous remains unclear and it is entirely possible that the clade represents an independent colonisation of the planktonic niche by a different group of benthic foraminifera.

The extant nonspinose macroperforate lineages are the result of a radiation in the last 30 million years (review in^63^). The monophyly of the Neogloboquadrinidae is strongly supported in the fossil record,^64^ the well documented lineage leading to *Globorotalia inflata* is clearly distinct from the Neogloboquadrinidae.^65^ The common origin of these lineages in SSU rDNA trees (Figs. 3, 4) receives little support (Table 4), and the preferred ML topology could be erroneous. There is equally ample fossil evidence for sister relationships between *N. incompta—pachyderma* and *N. dutertrei—Pulleniatina obliquiloculata*.^64^ These relationships are only weakly supported in our analyses as well as in all previous manual-alignment based analyses (Table 4; Additional file 3); they appear to be better resolved in taxonomically reduced datasets, in particular when the long-branching *N. incompta* is not included.^61^ Such eclectic sampling obviously cannot solve the issue of the phylogeny of the foraminifera; it can only be used to discuss specific relationships within clades. Several alternative interpretations of the fossil record exist to explain the relationships within the modern genus *Globorotalia*,^64^,^66^ but the genus is generally considered monophyletic with a common ancestor in the Oligocene around 35–30 million years ago. As in the case of *Neogloboquadrina*, this cannot be supported based on SSU rDNA data to date (Fig. 4; Additional file 3).

The spinose condition in planktonic foraminifera evolved within the genus *Eoglobigerina* in less than 100,000 years after the Cretaceous-Tertiary extinction event some 65 million years ago.^59^,^67^ An analysis of the fossil record following the initial radiation of the spinose taxa indicates that all subsequent lineages of spinose planktonic foraminifera with bilamellar shells (Table 1) can be linked to this one common ancestor.^59^,^60^,^64^ The origin of the extant family Hastigerinidae possessing monolamellar shells (Table 1), and represented by *H. pelagica* herein (Figs. 3, 5), remains unknown. Earlier attempts to ally *Hastigerina* with *Globigerinella siphonifera* on the basis of similarities in spine architecture have been shown to be misleading.^67^,^68^ In comparison to all other planktonic foraminifera, the monolamellar shells of both *Hastigerina* and *Hastigerinella* are extremely fragile and often partially resorbed during reproduction. As a result, they are only rarely preserved in marine sediments (a questionable report of *H. pelagica* is from the Miocene <10 million years ago)^64^ and the fossil record therefore bears little further evidence on their phylogenetic position. However, several extinct, fragile mono-lamellar taxa are known from the early Cainozoic, but no *H. pelagica* or any other monolamellar spinose species have been observed in the sediment. Given the position of *H. pelagica* in SSU rDNA trees (Figs. 3, 5), one could even speculate that this species might represent the latest colonisation of the planktonic niche from a completely different group of benthic foraminifera.

Within the spinose species, the sister relationships *Globigerina bulloides—G. falconensis*, *Globigerinoides ruber—G. conglobatus* and *Globigerinoides sacculifer—Orbulina universa* (Figs. 3, 5; Table 4; Additional file 3) are in agreement with the fossil record and largely congruent with former SSU rDNA phylogenies (Table 4).^3^,^7^,^9^,^11^,^64^ Furthermore, the *Globigerinoides-Orbulina* clade (Figs. 3, 5) is characterized by several potential morphological synapomorphies (supplementary apertures along the spiral suture, modifications of the last chamber) and the fossil record can be interpreted in favour of its monophyly.^64^ The *Turborotalita* lineage can be traced to the Eocene, at least 45 million years ago,^60^ and therefore it should have diverged closer to the root of the spinose subtree. Here, we found no unambiguous support for the placement of *T. quinqueloba* as sister group of *Globigerina falconensis* and/or *G. bulloides* and thus no evidence for an actual conflict between molecular and palaeomorphological data.^9^,^11^

The origin of the *Globigerinella siphonifera* lineage is not resolved in the fossil record. Based on its wall texture and the morphology of the first representatives of the lineage, it appears more closely related to *Globigerina* than *Globigerinoides*.^64^ In analogy to *Hastigerina*, neither the fossil evidence nor the molecular (SSU rDNA) support is sufficient to unambiguously identify the sister clade to this species. In contrast to other ‘deep’ divergences, the according bipartition received only moderate support under ML (clustalw-based none; Figs. 3, 5) and diminishing support under MP (details not shown, Additional file 3).

## Conclusion

As depicted in Figure 3, SSU rDNA sequences extracted from morphologically defined species of planktonic foraminifera can be supported as clades (monophyla) by phylogenetic analysis of *complete* fragments of SSU rDNA despite the large divergence and length polymorphism in the expansion segments. Using a reproducible approach based on automated alignments without a priori filtering of nucleotides, we were able to infer several phylogenetic relationships, which obtain significant support from bootstrap analyses of all underlying data matrices (Figs. 3–5, Tables 3, 4; Additional files 2, 3). Thus, these relationships are supported *independently* of alignment ambiguity. The newly reported relationships are at least as congruent with the evidence from the fossil record as those inferred from time-consuming manual alignments after manual exclusion of not unambiguously alignable regions. This indicates that the need to establish nucleotide homology is not the most important obstacle when exploring the phylogenetic structure of the SSU rDNA in planktonic foraminifera. In our multiple analysis approach, important clades were recovered with much less effort than before, and in many cases, with higher support. Importantly, the lower alignment effort enabled us to include *all* available SSU rDNA sequences of planktonic foraminifers in the analyses; to the best of our knowledge, this was done for the first time in the present study.

Regarding the phylogenetic backbone of the planktonic foraminifera tree, many relationships remained ambiguous. The clarification of the relationships within the groups of nonspinose macroperforate planktonic foraminifera and between spinose subclades requires a reinvestigation of the fossil (sediment) record, a re-evaluation of the morphological traits uniting these clades, and additional molecular data covering all known planktonic species. Such combination of molecular, morphological and fossil data has the potential to provide an unprecedented level of understanding of the evolutionary unfolding within planktonic foraminifera.

It is apparent that future efforts in reconstructing the phylogeny of planktonic or other foraminifera with large divergences in SSU rDNA sequences should focus on exploring the effect of distinct alignments on the phylogenetic signal from the SSU rDNA without prior subjective filtering of the data. The same recommendation is likely to apply to other organisms and other alignment-ambiguous loci.^24^,^25^ Use of up-to-date versions of several alignment programs under default values appears reasonable, while at least some potential artefacts as caused by, e.g. incompletely known sequences can be recognized by automated filtering using the comparison with previous information on probable taxonomic relationships.

## Additional Files

Additional file 1—Sequence list List of sequences newly obtained in the current study, including accession numbers and affiliations to morphospecies.

Additional file 2—Alignments and phylogenetic trees Contains all alignments in FASTA and all phylogenetic trees in Newick format inferred in the course of the study. The trees are not collapsed and appropriately annotated. The pseudocharacter matrix for the comparison with the current taxonomy is also contained, as well as the substitution matrix used for running poa and poaglo.

Additional file 3—Bootstrap support under ML and MP of selected bipartitions Comparative list of bootstrap support under ML and MP based on the six selected alignments; along with a qualitative rating of the listed bipartitions, and according phylogenetic relationships.

## Figures and Tables

**Table 1.** Species of planktonic foraminifers. A list of all planktonic foraminifera species included in this study; and their representation by SSU rDNA data in public databases and newly assembled data.

**Species**	**SSU data available§**	**New data added**
**Microperforate clade (** = **Candeinidae Saito and Thompson 1982)**		
*Candeina nitida* d’Orbigny 1839	No	No
*Globigerinita glutinata* (Egger 1893)	Yes	Yes†
*G. minuta* (Natland 1938)	No	No
*G. uvula* (Ehrenberg 1861)	Singleton	Yes†
*Tenuitella fleisheri* Li 1987	No	No
*T. iota* (Parker 1954)	No	No
*T. parkerae* (Brönnimann and Resig 1971)	No	No
**Nonspinose clade (** = **Globorotaliidae Cushman 1927)**		
*Berggrenia pumilio* (Parker 1962)	No	No
*Globoquadrina conglomerata* (Schwager 1866)	No	No
*Globorotalia anfracta* (Parker 1967)	No	No
*G. cavernula* Bé 1967	No	No
*G. crassaformis* (Galloway and Wissler 1927)	Singleton*	No
*G. hirsuta* (d’Orbigny 1839)	Singleton	Yes
*G. inflata* (d’Orbigny 1839)	Singleton	Yes
*G. menardii* (d’Orbingy 1826)	Yes	No
*G. scitula* (Brady 1882)	No	No‡
*G. truncatulinoides* (d’Orbigny 1839)	Yes	Yes†
*G. theyeri* Fleisher 1974	No	No
*G. tumida* (Brady 1877)	No	No
*G. ungulata* Bermudez 1960	No	No
*Globorotaloides hexagonus* (Natland 1938)	No	No
*Neogloboquadrina dutertrei* (d’Orbingy 1826)	Yes	No
*N. incompta* (Cifelli 1961)	Yes	Yes
*N. pachyderma* (Ehrenberg 1861)	Yes	No
*Pulleniatina obliquiloculata* (Parker and Jones 1862)	Yes	No
**Spinose bilamellar clade (** = **Globigerinidae Carpenter, Parker and Jones 1876)**		
*Beela digitata* (Brady 1879)	No	No
*Globigerina bulloides* d’Orbigny 1826	Yes	No
*G. falconensis* Blow 1959	Yes	No
*Globigerinella adamsi* (Banner and Blow 1959)	No	No
*G. calida* (Parker 1962)	Singleton*	No
*G. siphonifera* (d’Orbigny 1839)	Yes	Yes
*Globigerinoides conglobatus* (Brady 1879)	Yes	No
*G. ruber* (d’Orbigny 1839)	Yes, biphyletic	No
*G. sacculifer* (Brady 1877)	Yes	No
*Globoturborotalita rubescens* Hofker 1956	No	No
*G. tenella* (Parker 1958)	No	No
*Orbulina universa* d’Orbigny 1839	Yes	No
*Sphaerodinella dehiscens* (Parker and Jones 1865)	No	No
*Turborotalita clarckei* (Roegl and Bolli 1973)	No	No
*T. humilis* (Brady 1884)	No	No
*T. quinqueloba* (Natland 1938)	Yes	No
**Spinose monolammelar clade (**= **Hastigerinidae Saito and Thompson 1976)**		
*Hastigerina pelagica* (d’Orbigny 1893)	Singleton	Yes†
*Hastigerinella digitata* (Rhumbler 1911)	No	No
*Orcadia (Hastigerinella) riedeli* (Roegl and Bolli 1973)	No	No

* These singletons are possibly not representative for the assigned species.

† The new data revealed new sequence (sub)types.

‡ The new data includes sequences from a globorotaliid specimen, which may be *G. scitula* or not.

§ Available in public databases at the time of data mining (October 2008). A SSU rDNA sequence of *C. nitida* is available since the end of 2008.^69^

**Table 2.** Features of the alignments and phylogenetic trees. This table lists features of the eleven sequence alignments constructed and the resulting phylogenetic trees. The entire alignment length is shown. For the resulting best ML trees, the final estimate for the alpha value of the gamma distribution and the log likelihood of the best tree are shown, as well as the sum of the Robinson-Foulds (RF) distances of each tree to the other nine trees and the agreement with the affiliation of sequences to morphospecies (T-score; lower scores indicate better agreement). Note that the likelihood of the best tree cannot directly be used to select the best alignment, because common ML functions as those implemented in RAxML do not consider gaps.

**Alignment software**	**Alignment length**	**Final alpha value**	**Highest Log likelihood**	**Sum of RF distances to other trees**	**T-score**
**CLUSTALW**	**1384**	**0.93969**	**3,582,498,665**	**3496**	**23**
CLWOPT	1557	0.97349	−3,598,746,746	3416	25
EINSI	1786	0.48367	−3,012,840,593	3194	23
GINSI	1837	0.48314	−2,849,473,664	3206	23
**KALIGN**	**1905**	**0.62220**	−**3,251,648,372**	**3482**	**23**
LINSI	1751	0.53379	−3,069,451,219	3226	23
**MAFFT**	**1965**	**0.54546**	−**3,075,848,970**	**3032**	**23**
MUSCLE	2192	0.82643	−5,422,632,153	4126	25
**NRALIGN**	**1797**	**0.75213**	−**4,765,997,803**	**3772**	**23**
**POA**	1856	0.60630	−3,203,410,297	3356	23
**POAGLO**	**1840**	**0.67321**	−**3,506,284,042**	**3374**	**23**

Alignments considerd for Results and Discussion in bold font.

**Table 3.** Support of morphotaxa under parsimony. ML bootstrap support (see also Fig. 3) is included for comparison. *Hastigerina pelagica* is, in addition to the known problematic case of *Globigerinoides ruber* (see text) the only morphotaxon that receives no sufficient support.

**Alignment used**	**Nonparametric bootstrap support under ML**						**Nonparametric bootstrap support under MP**										
	**CLUSTALW**	**KALIGN**	**MAFFT**	**NRALIGN**	**POA**	**POAGLO**	**CLWOPT**	**CLUSTALW**	**EINSI**	**GINSI**	**KALIGN**	**LINSI**	**MAFFT**	**MUSCLE**	**NRALIGN**	**POA**	**POAGLO**
**Microperforate species**																	
*Globigerinita glutinata*	100	19	91	98	95	97	0	100	100	100	100	100	100	1	100	100	100
*G. uvula*	100	100	100	96	60	89	100	100	77	80	100	95	96	99	94	26	59
**Macroperforate nonspinose species**																	
*Globorotalia hirsuta*	98	99	99	97	99	94	100	100	100	100	100	100	100	100	100	100	100
*G. inflata*	99	98	100	100	100	94	100	100	100	100	100	100	100	100	100	100	100
*G. menardii*	100	100	100	100	100	100	100	100	100	100	100	100	100	100	100	100	100
*G. truncatulinoides*	100	100	100	100	100	100	100	100	100	100	99	100	100	100	100	100	100
*Neogloboquadrina dutertrei*	100	100	94	100	100	100	54	100	100	100	100	72	100	0	100	100	100
*N. incompta*	100	100	99	98	86	99	100	100	100	100	100	100	100	64	100	100	100
*N. pachyderma*	100	100	100	100	100	100	100	100	100	100	100	100	100	100	100	100	100
*Pulleniatina obliquiloculata*	99	98	99	100	98	100	100	100	100	100	100	100	100	100	100	100	100
**Spinose species**																	
*Globigerina bulloides*	93	99	100	98	100	100	100	100	100	100	100	100	100	100	100	100	100
*G. falconensis*	100	100	100	100	100	100	100	100	100	100	100	100	100	100	100	100	100
*Globigerinella siphonifera*	100	89	100	99	100	100	100	100	100	100	12	100	100	98	100	100	100
*Globigerinoides ruber* s.str.	100	100	100	100	100	100	100	100	100	100	100	100	100	100	100	100	100
*G. conglobatus* s.l.	99	97	95	94	90	82	100	100	95	99	100	100	100	81	90	99	99
*G. sacculifer*	100	100	100	100	100	100	100	100	100	100	100	100	100	100	100	100	100
*Hastigerina pelagica*	4	0	38	9	88	68	1	4	5	12	2	8	13	0	6	24	31
*Orbulina universa*	100	100	100	94	86	85	100	99	96	87	100	93	100	61	86	91	93
*Turborotalia quinqueloba*	100	100	100	93	100	100	100	100	100	100	100	100	100	90	99	100	100

Moderate and low support values are highlighted.

**Table 4.** Support for selected phylogenetic scenarios. Comparison of our multiple analysis results (Fig. 3; Additional files 2, 3; BS under ML and MP) with eight previous manual-alignment based phylogenetic reconstructions in terms of the statistical support for relationships that appear to be consistently resolved in the fossil record of planktonic foraminifera. Values of support for each node are given where the respective study have identified the node as the dominant signal; “no” indicates analyses where an alternative topology has been preferred and “N/A” indicates analyses where some of the constituent species of the clade above the node have not been included.

	**Microperforate clade**	**Macroperforate clade**	***G. truncatulinoides—G. hirsuta*clade**	***Neogloboquadrina—Pulleniatina*clade**	***P. obliquiloculata—N. dutertrei*clade**	***N. pachyderma—N. incompta*clade**	**Spinose clade**	***G. bulloides—G. falconensis*clade**	***G. ruber—G. conglobatus*clade**	***O. universa—G. sacculifer*clade**	***Globigerinoides—O. universa*clade**
Darling et al^2^	N/A	No	N/A	N/A	N/A	N/A	(No)*	N/A	99	82	87
De Vargas et al^3^	N/A	46/41/73	N/A	N/A	N/A	N/A	No/58/51	N/A	91/100/100	No	No
De Vargas and Pawlowski^5^	N/A	N/A	47	N/A	N/A	N/A	(81)*	N/A	100	<50	No
Darling et al^7^	N/A	No	N/A	N/A	N/A	N/A	(57)*	N/A	100	47	No
Darling et al^9^	N/A	(76)‡	N/A	N/A	N/A	Unresolved	(86)*	N/A	99	<50	Unresolved
Stewart et al^11^	Unresolved	(69)§	N/A	N/A	N/A	N/A	(88)*	No	98	<50	No
Darling et al^54^	Unresolved	<70	N/A	N/A	78 (?)	Unresolved	<70	N/A	100	<70	No
Ujiié et al^69^	1.00/100	0.88/80	No	No	Unresolved	N/A	0.87/52	N/A	1.0/100	0.83/80	Unresolved
**Multiple analysis**											
**BS_ML_**	100–59	82–30 (10†)	78–2	39–5	91–0	30–5	100–37	94–56	100–99	100–83 (0†)	100–32
**BS_MP_**	100–52	20–0	34–0	7–0	99–0	14–0	61–22 (0†)	100–56	100	99–64 (0†)	66–12

* These studies did not include the phylogenetically challenging taxon *Hastigerina pelagica.*

† Based on the KALIGN-generated alignment (see text).

‡ No *Globorotalia* species included.

§ Only two close relatives included.

## Abbreviations

BS_ML_: bootstrap support under ML
BS_MP_: bootstrap support under MP
ML: maximum likelihood
MP: maximum parsimony.
