# Supplementary material for: Using the Multiple Analysis Approach to Reconstruct Phylogenetic Relationships among Planktonic Foraminifera from Highly Divergent and Length-polymorphic SSU rDNA Sequences
Source: Bioinform Biol Insights. 2009 Nov 11;3:155–77. doi: 10.4137/bbi.s3334 (PMC2808177; doi:10.4137/bbi.s3334)
Supplement: Additional file 1—Sequence list [file Additional_File_1.zip › Additional_File_3.pdf]

Taxonomic unit / phylogenetic relationship — Alignment used	Nonparametric bootstrap support under ML: ClustalW	KALIGN	MAFFT	NRALIGN	POA	POAGLO	Nonparametric bootstrap support under MP: ClustalW	KALIGN	MAFFT	NRALIGN	POA	POAGLO
Split between microperforate and others	100	99	99	97	59	82	100	100	100	97	52	67
G. glutinata	100	19	91	98	95	97	100	100	100	100	100	100
G. uvula	100	100	100	96	60	89	100	100	96	94	26	59
Split between macroperforate nonspinose and others	70	10	30	72	82	54	1	0	16	8	20	16
Genus *Globorotalia*	—- Not recognized as clade —-											
G. hirsuta	98	99	99	97	99	94	100	100	100	100	100	100
G. inflata[a]	99	98	100	100	100	94	100	100	100	100	100	100
G. menardii	100	100	100	100	100	100	100	100	100	100	100	100
Globorotalia ?sp.	100	100	100	100	100	100	100	100	100	100	100	100
G. truncatulinoides	100	100	100	100	100	100	100	99	100	100	100	100
Genus *Neogloboquadrina*[b]	7	5	33	5	39	35	0	0	1	1	2	7
N. dutertrei + Pulleniatina obliquiloculata	0	43	35	0	66	91	0	71	64	12	99	98
N. dutertrei	100	100	94	100	100	100	100	100	100	100	100	100
P. obliquiloculata	100	100	99	98	86	99	100	100	100	100	100	100
Former *N. pachyderma*	5	7	17	30	22	28	3	0	1	14	4	4
N. incompta	100	100	100	100	100	100	100	100	100	100	100	100
N. pachyderma	99	98	99	100	98	100	100	100	100	100	100	100
Split between spinose and others	92	44	100	100	77	37	49	0	53	61	25	22
Hastigerina types placed as sister clades	4	0	38	9	88	68	4	2	13	6	24	31
Hastigerina types placed as grade	5	0	16	52	0	8	21	1	17	18	0	1
H. pelagica, known type	100	100	100	100	100	100	100	100	100	100	100	100
New *H. pelagica* type	100	100	100	100	100	100	100	100	100	100	100	100
All other spinose forming a clade	51	5	64	51	24	20	29	0	29	7	16	4
Globigerinella sister to *Globigerinoides+Orbulina*	3	69	86	53	67	56	3	5	26	18	26	26
Globigerinella sister to *Globigerina+Turboritalia*	1	0	0	0	14	2	2	0	5	1	20	2
Globigerinella sister to remaining spinose	59	0	0	2	0	0	24	4	3	1	0	0
Globigerinella siphonifera clade	100	89	100	99	100	100	100	12	100	100	100	100
G. siphonifera type I	100	99	100	100	100	100	100	100	100	99	100	100
G. siphonifera type I sister to types II+III and IV	0	72	95	99	13	89	88	0	94	98	21	51
Clade with *G. siphonifera* types II and III	0	48	98	98	40	0	85	0	98	99	78	69
G. siphonifera type II[c]	0	26	79	95	30	0	38	0	32	81	34	27
G. siphonifera type III	95	93	84	97	85	84	85	92	68	79	94	95
G. siphonifera type IV	100	99	100	100	100	99	100	100	100	100	100	100
Globigerinoides-Orbulina clade	100	79	34	32	92	60	66	28	17	12	63	40
Genus *Globigerinoides*	0	100	6	0	3	0	0	100	6	6	24	0
G. ruber-conglobatus clade	100	100	100	100	100	100	100	100	100	100	100	100
G. ruber s.str.	100	100	100	100	100	100	100	100	100	100	100	100
G. conglobatus s.l.	99	97	95	94	90	82	100	100	100	90	99	99
G. sacculifer	100	100	100	100	100	100	100	100	100	100	100	100
G. sacculifer-Orbulina clade	100	0	83	98	95	100	99	0	64	86	64	99
Orbulina clade	100	100	100	94	86	85	99	100	100	86	91	93
Globigerina-Turborotalita clade	44	12	79	51	65	35	13	9	18	37	57	22
Globigerina monophyletic	94	90	77	80	56	88	100	100	85	53	53	68
G. bulloides	93	99	100	98	100	100	100	100	100	100	100	100
G. falconensis	100	100	100	100	100	100	100	100	100	100	100	100
Turborotalia quinqueloba	100	100	100	93	100	100	100	100	100	99	100	100

[a] Including the accession labelled as *Globorotalia crassaformis*

[b] Including *P. obliquiloculata*

[c] Including the accession labelled as *Globigerinella calida*

- Green: Well-supported by nonparametric bootstrapping under ML and MP
- Yellow: Moderately to well-supported
- Orange: Not unambiguously supported
- Red: Not supported
- Black frame: Differently supported under ML and MP
